# Perceptions, behaviours, barriers and needs of evidence-based medicine in primary care in Beijing: a qualitative study

**DOI:** 10.1186/s12875-019-1062-0

**Published:** 2019-12-06

**Authors:** Yali Zhao, Xuexue Zhao, Yanli Liu, Yun Wei, Guanghui Jin, Shuang Shao, Xiaoqin Lu

**Affiliations:** 1 School of General Practice and Continuing Education, Capital Medical University, No. 10, Xitoutiao, You An Men Wai, Fengtai District, Beijing, 100069 China; 2 Department of General Practice, Beijing Tiantan Hospital, Capital Medical University, Beijing, China

**Keywords:** Views, Evidence-based medicine, Qualitative research, Primary care

## Abstract

**Background:**

Evidence-based medicine (EBM) is gradually being recognized worldwide as an important clinical skill and plays an important role in health care. Although the concept has successfully spread in the health care field, EBM still has not been widely incorporated into clinical decisions in primary care due to potential barriers. This study aimed to explore the views, experiences and obstacles of general practitioners (GPs) regarding the use EBM in their daily clinical practices in Beijing.

**Methods:**

We performed a qualitative study with GP focus groups. Thirty-two GPs working in 26 community health service centres in 7 districts in Beijing were recruited. Four focus group sessions with 32 GPs were conducted in a meeting room at the Capital Medical University from January to February in 2018 in Beijing. All sessions were audio-recorded, transcribed and analysed for themes using an inductive content analysis approach.

**Results:**

GPs believed that EBM could help them enhance the quality of their clinical practice. The most common EBM behaviour of GPs was making clinical decisions according to guidelines. The barriers that limited the implementation of EBM were patients’ poor compliance, lack of time, lack of resources, inadequate skills or knowledge, and guideline production problems. The first need for GPs was to participate in training to enhance their skills in practising EBM.

**Conclusions:**

To practise EBM in general practice, integrated interventions of different levels need to be developed, including enhancing GPs’ communication skill and professional competency, training GPs on the implementation of EBM, employing more staff to reduce GPs’ workloads, providing adequate resource support, and developing evidence-based clinical guidelines for GPs.

## Background

Evidence-based medicine (EBM) is ‘the conscientious, explicit and judicious use of current best evidence, in combination with the physician’s clinical expertise and the preferences and situation of the patient in making decisions about the care of individual patients’ [1, 2]. In this definition, three key elements are identified: the evidence; the physician’s basic skills, personal experience and expertise; and the patient’s condition and preferences [3]. Since it was introduced in the early 1990s, EBM has been gradually recognized worldwide as an important clinical skill that plays an important role in health care [4]. It is assumed that the application of EBM will lead to improvements in health care provision [5]. A prerequisite for practising EBM is access to the current best evidence, which requires physicians to have specific knowledge and skills and a positive attitude towards EBM [3, 6]. For GPs, the best evidence will usually be the guidelines. Working according to guidelines is regarded as one of the main indicators of EBM in primary care [7]. When guidelines cannot be applied, the current best evidence should be derived, preferably using the five EBM steps (ask, access/acquire, appraise, apply, audit) [8]. Although there is no doubt that the concept of EBM has been successfully disseminated in the field of health care, EBM still has not been widely incorporated into clinical practice due to criticism and controversy [9]. A systematic review [10] found that the most common barriers to the implementation of EBM were related to research barriers, lack of resources, lack of time, inadequate skills, inadequate access, lack of knowledge and financial barriers.

China launched national health system reforms in 2009 with the aim of strengthening the primary healthcare system [11]. High quality primary health care plays a vital role in achieving universal health coverage and meeting growing health needs [12]. Adequate and competent GPs are essential for a strong primary care system [13]. The major responsibilities of GPs are to provide common medical services and basic public health services, including diseases diagnose and treating, health education and consulting, chronic disease management and rehabilitation services [11]. To develop professional competence of GPs, China promulgated a plan to standardise general practice postgraduate residency training program in 2011, which emphasized a 3-year general practice residency training after 5 years of undergraduate clinical medicine study (5 + 3 model), like doctors in other clinical specialties [13]. Meanwhile, another model involves retraining the majority of less-educated physicians who currently work in local CHS institutions, and transforming them into qualified GPs [14]. To less-educated GPs, who currently work in community and want to get better personal development, some medical universities provided on-the-job general practice master’s degree training programmes for them during their part-time.

At present, China is yet faces a critical shortage of qualified GPs. In 2017, China’s total licensed doctors are 2.8 million, of which 252,717 are GPs. The number of GPs per 10 thousand inhabitants is 1.82 [15]. A study found that the largest amount of consultations per day (eight hours work time) was 91.0 for a chief GP in community clinic in Beijing due to shortage of GPs [16]. CHS institutions in China cannot attract enough GPs due to low salary, low social status, limited career opportunities, and a lack of the public trust [14]. Doctors, especially male doctors, with a higher medical degree are more likely to find a job in a higher-level hospital for better future career progression and higher income [17]. In 2017, of licensed doctors in CHS centres in China, only 49.1% were 5 year or over medical educational background, and female doctors accounted for 56.3% [15]. A study [18] found that 73.1% of 474 participating GPs from Beijing urban community were females.

EBM was introduced in mainland China over two decades ago. The organized dissemination of EBM aims to solve clinical problems and promote the efficacy of clinical medicine, applied clinical practice and health policy decision making [19]. However, in primary care settings, few studies are available on GPs’ perceptions and attitudes towards EBM in China. EBM’s true impact in primary care is uncertain, and barriers to applying EBM are not clear. This study aims to explore the experiences and determine the barriers and needs of GPs regarding EBM based on GPs’ perceptions in China.

## Methods

### Sample

According to the inclusion criteria of participants listed in Table 1, we recruited 34 GPs from a list of general practice master’s degree candidates. These full-time GPs participated in on-the-job master’s degree training on their weekends at Capital Medical University in 2017. One member of our research team was responsible for conducting sampling. Two of the 34 GPs did not participate in the interview due to time constraints; finally, we interviewed 32 GPs. In this study, we use the term ‘GPs’ synonymously with that of the participants.

**Table 1:** The inclusion criteria for GPs

Inclusion criteria	(1) Work experience in the community clinic for 5 years or more, (2) employment at government-owned and -managed CHSCs^*^ in Beijing, and (3) an interest in the study.

^*^Community health service centres

### Design

We selected and interviewed a total of 32 GPs working in 26 CHS centres in 7 districts in Beijing, as shown in Table 2. Eight GPs were recruited for each group, and 4 focus groups were conducted in a meeting room of the Capital Medical University from January to February 2018.

**Table 2:** Sample GPs from 7 districts in Beijing

Districts	Chao yang	Feng tai	Hai dian	Xi cheng	Dong cheng	Chang ping	Tong zhou	total
No. of involved CHS centres*	10	3	5	2	1	3	2	26
No. of GPs from involved centres	**12**	**5**	**7**	**2**	**1**	**3**	**2**	**32**

*: The CHSCs in our study are government-own and -managed organizations in Beijing. These figures were obtained from the Beijing community health service network, 2018

### Data collection

Focus group sessions facilitated by a moderator lasted 60–90 min and were audio-taped with the permission of the GPs. Before the interview, the moderator explained the reason and value for conducting the research to the GPs. The point of information saturation was reached when the fourth focus group was completed. The GPs could stop the interview at any stage without giving a reason. The moderator was an expert in general practice and was very familiar with EBM. One researcher took notes on a computer during the meetings. To standardize the interview process, the interviewers received pre-study training in qualitative interviews and analysis, which was organized by the researchers. No one else was present besides the GPs and researchers during the interviews. The interviewers did not contact the GPs before the study.

The ages of the 32 GPs ranged from 29 to 48 years old (mean age = 36.88 ± 5.01 years old), and their working experience ranged from 5 to 26 years (mean working experience =12.31 ± 6.12 years), as shown in Table 3. The interview questions covered issues about the GPs’ understanding of EBM, their clinical behaviour regarding EBM, existing barriers to implementing EBM, and their needs for implementing EBM after pilot testing (see Additional file 1).

**Table 3:** Demographic characteristics of GPs

Category	Subcategory	N
Gender	female	28
	male	4
Age	≤29	2
	30–39	23
	≥40	7
Work experience years	5–9	10
	10–19	18
	≥20	4
Professional positions	senior-level title	3
	middle-level title	27
	junior-level title	2

### Data analyses

We used an inductive content analysis approach [20] to analyse the data between March and April 2018. Summaries of the 4 focus groups were created according to the reviewed audiotapes and notes. Two members of the research team read the contents from the four focus groups repeatedly and then summarized and coded transcripts independently by reviewing the audio tapes and the notes. After coding, lists of categories were created under higher order headings. Finally, the main categories and subcategories were formulated and grouped together by the abstraction process. The differences in codes, category creation, and the abstraction process were resolved by interactive discussions among co-researchers, which ensured the representativeness of the emerging themes and the internal validity of the content analysis.

## Results

Four dominant themes were identified from the insights of the GPs: perceptions of EBM, behaviours of practising EBM, barriers to practising EBM, and needs for practising EBM.

### Perceptions of EBM

The majority of the GPs in our sample did not fully understand the concept of EBM, but most of them agreed that EBM could help them effectively resolve clinical problems, enhance the quality of clinical practice, and improve patients’ health outcomes.

“*I think EBM is retrieving and using evidence to resolve clinical questions and guide clinical practice.*”(GP19) *“I… I don’t know clearly about the concept of EBM.”* (GP10).

*“Practising EBM can improve my professional capacity.”* (GP18) *“It (EBM) helps GPs correctly make clinical decisions by providing decision recommendations and good evidence.”* (GP11).

### Behaviours of practising EBM

The GPs EBM behaviours mainly focused on referring to clinical practice guidelines for chronic disease management and adopting advice from specialists or colleagues. Some of the GPs searched medical specialty information from mobile medical apps and network databases.

*“To me, my evidence-based practice is using clinical guidelines for chronic diseases, mainly hypertension, diabetes, coronary heart disease and stroke.”* (GP1).

*“Specialists from tertiary hospitals see patients in our clinic regularly. I can get new evidence and effective advice about diagnoses and treatments from these specialists.”* (GP8) *“When confronted by a clinical problem, I will discuss with my colleagues or consult senior doctors to get advice.”* (GP5).

*“Now, I often seek medical information and evidence from mobile medical apps....um... such as DingXiangYuan, HaoYiSheng, SpringRain, and MedSci. I benefit a lot from these apps.”* (GP2) *“Official WeChat accounts for medicine provide plenty of useful information for my patients.”* (GP24) *“When I encounter clinical problems, I search literature from the CNKI (China Knowledge Resource Integrated Database) or WanFang database after payment.”* (GP16).

### Barriers to practising EBM

The majority of GPs noted that there were many barriers to practising EBM. These were categorized into patients’ poor compliance, lack of time, lack of resources, inadequate skills or knowledge, and guideline production problems. These barriers caused GPs not to implement EBM effectively in patient encounters.

#### Patients’ poor compliance

Regarding patients, the main concerns of the GPs were focused on patients’ poor compliance due to distrust of the GPs, and lack of correct health information.

*“Many patients don’t trust GPs; they would rather accept therapy plans created by doctors from tertiary hospitals. When I try to adjust the treatment plans based on evidence and the patient’s condition, the patient generally refuses my suggestion.”* (GP1).

*“Before seeing a doctor, some patients search inadequate medical information on the internet. When communicating with a GP, they often show the GP the information and question the GP’s clinical decision.”* (GP3).

#### Lack of time

Regarding time, insufficient time to practice EBM owing to work overload was commonly mentioned by most GPs.

*“My time is fragmented by trivial and heavy work, (which means) I have no more time to study and apply EBM.”* (GP16) *“I need to see 70-80 patients in the clinic every day. It is impossible to take extra time to explain study evidence to a patient; it is very time consuming.”* (GP9).

#### Lack of resources

Regarding lack of resources, a lack of medical databases and equipment were named as the main hindrances for GPs to acquire the latest medical information and evidence.

*“Many research references need be retrieved and downloaded from all kinds of databases. However, none of the databases have been purchased by our hospital for the staff.”* (GP16).

*“Some examination facilities or clinical drugs recommended in guidelines are not available in our community hospital.”* (GP14).

#### Inadequate skills or knowledge

Regarding inadequate skills or knowledge, some of the GPs admitted that they lacked the capability to search and evaluate evidence. Moreover, they did not know how to interpret study evidence and consider the applicability to their individual patients.

*“I don’t know how to seek guidelines from internet databases.”* (GP8) *“It is too difficult for me to assess the quality of study evidence...”* (GP6).

*“I can’t judge whether study evidence is applicable to my individual patients.”* (GP5).

#### Guideline production problems

Regarding guideline production problems, the GPs admitted that the limited relevance of guidelines to clinical practice hindered its usability in specific clinical scenarios. Additionally, they were often puzzled by uncertain and changing evidence in guidelines.

*“Study results in many guidelines report the generality of evidence, but they are less likely to answer individualized questions.”* (GP15) *“Most of the clinical guidelines are developed by specialist working groups from specialized hospitals and are inapplicable to the breadth of practice in community hospitals.”* (GP18).

*“I feel clinical evidence always changes. For example, according to a guideline, I provided some treatment recommendations for my patients for quite a time. However, I found that the evidence changed when the guideline was updated…So… I am confused and don’t know how to explain that to the patients.”* (GP26).

### Needs for practising EBM

The main needs for practising EBM were focused on participating in training to enhance skills in practising EBM, resource support, and reducing workloads.

*“Adequate continuing EBM training should be provided and (the contents of training) should focus on the literature retrieval, EBM practice skills and the guidelines’ application in general practice.”*(GP21).

*“We need to obtain free medical information with full texts, so I’m eagerly looking forward to our hospital purchasing medication databases for us.”* (GP7) *“Guidelines for general practice should be developed based on experienced GPs’ participation and community data.”* (GP2).

*“The number of GPs is seriously inadequate, so we undertake a huge quantity of work. We hope more GPs can be employed to lessen our workload.”* (GP13).

## Discussion

The gap between knowledge production and its implications is the main reason for the performance difference in health care. The EBM approach is considered an important promoting factor to eliminate this gap and improve health care quality [10]. With the rapid growth in the volume of medical information and complex treatment procedures, the most valid evidence and recommendations should be widely available to primary care professionals practising EBM [4]. Our study provides recommendations for policy makers for the development of countermeasures by exploring the behaviours, barriers and needs of GPs practising EBM.

### GPs share a positive attitude towards EBM, and the most common EBM behaviour of GPs is making clinical decisions according to guidelines

In agreement with earlier findings [21, 22], the vast majority of the GPs in our study believed that working according to the principles of EBM in general practice could improve the quality of patient care and had positive attitudes towards EBM. All but two GPs in our study did not correctly understand the meaning of EBM, which revealed the importance and urgency of GPs participating in EBM to receive training and education.

In our study, the GPs reported that using guidelines and consulting specialists or colleagues were the top 2 behaviours applied in EBM, which is in concordance with a previous study [23]. An advantage of guidelines is that they help GPs to practise EBM effectively by saving time that would otherwise be required for searching for and critically appraising information [24]. Compared with other doctors, GPs were significantly more uncertain about the accessibility of and the evidence in guidelines [25]. In China, only 4.2% of the guidelines developed were aimed at GPs, and the utilization of guidelines in primary care is deficient. An increasing number of GPs require guidelines for chronic disease management, which should be considered a priority [26].

In addition to guidelines, consulting specialists were valued as both sources of information and interpreters of new evidence [27]. An important reason for GPs to consult specialists or colleagues is probably because specialists or colleagues provide answers very quickly and the answer can be directly utilized [23]. However, with this approach, GPs should be particularly attentive to whether the opinions of specialists or colleagues are relevant to their patients [27].

Seeking information from electronic clinical sources was the GPs’ third most common EBM behaviour after following guidelines and consulting specialists/colleagues. The majority of the GPs in our study commented that they had access to the internet in their workplace, but accessing electronic databases was difficult. Among electronic sources, smartphones were frequently used by GPs to seek medical information to resolve clinical questions. With the popularity of the internet, an increasing number of GPs used the internet to search for clinical information, and smartphone technology has been considered to be supplement to medical information seeking [28]. However, it was not always easy for GPs to retrieve medical information precisely by smartphone.

### GPs encounter many obstacles when practising EBM; the patients’ poor compliance is most frequently noted. Countermeasures should be taken to resolve these obstacles and meet the needs of GPs

The calls for incorporating EBM into clinical practice remain challenging due to the obstruction by many barriers [29]. In our study, the GPs voiced that some barriers limited their EBM practice behaviours.

In our study, patients’ poor compliance was the first problem. In China, patients still prefer to go to see specialists in a hospital instead of consulting GPs when they fall ill [30]. Poor doctor-patient relationships, brief consultations and low levels of trust in China have resulted in patients’ poor compliance [31]. Enhancing GPs’ communication skill and professional competency by continuous training is a main measure to improve more patients’ compliance with GPs’ advice.

Time availability is an important factor for applying EBM. Health care providers’ heavy workloads are the main reason for insufficient time [32]. In our study, the lack of time to search for, study and apply evidence in the job setting owing to GPs’ busy clinic work was the second barrier. Patients with stable chronic disease conditions are the main target population who visit GPs for prescriptions and primary care in China. An investigation [16] confirmed that 81.0% of all visits in the GP clinic in Beijing were for specific prescriptions or were chronic patients coming for prescription renewal, and the median consultation length of all visits was 2 min when there were many patients waiting for consultation. These disproportionate visits for prescription renewal mean GPs have little chance to use EBM. Moreover, a lack of ability to allocate time reasonably is also believed to constitute a barrier [24]. Training more GPs through 5 + 3 model is a main solution. The government should re-employ competent retired GPs back in primary care by increasing salary. Meanwhile, running ‘time management’ workshops can be useful to train staff in managing time. Furthermore, a significant amount of evidence summarized in clinical guidelines for general practice can help GPs to reduce the amount of time needed to answer clinical questions.

Compared with tertiary hospitals, community hospitals are equipped with less electronic information resources. In our study, GPs had access to the internet in their workplace. However, their access to electronic databases was hindered, and a lack of resources was the most common barrier to the implementation of guidelines [10]. The results of our study confirm that many recommendations from guidelines cannot be implemented by GPs due to limited facilities and inadequate medicine in primary care. Practitioners wanted more reliable and more relevant documents for daily practice. To solve this problem, many interventions could be performed. Providing adequate resources such as the development and popularization of user-tailored medical search tools and increasing the accessibility to medication databases during GP consultations are the first measures of governmental support [33]. In addition, websites with already selected resources could increase GPs use of the internet for medical information seeking [34]. Finally, proper resource allocation should be planned when policymakers and managers make decisions.

The results of our study indicate that the GPs did not have adequate knowledge and skills for searching for, evaluating and applying evidence. A survey found that most GPs did not have any formal training in searching databases and were unaware of many internet resources [35]. Enhancing knowledge of and skills for practising EBM is the first need for GPs in our study. Therefore, multiple interventions should be available for GPs. These interventions involve using web-based tools, participating in long-term EBM curricula, meeting regularly with colleagues experienced in information retrieval, and actively conducting clinical librarian and EBM resident rotations, all of which have been shown to be effective in reducing specific barriers to practising EBM [36]. Annual EBM knowledge exams and quality of care monitoring are effective strategies for organizations to sustain an enhanced EBM practice [37]. Furthermore, monthly training related to guidelines organized by authoritative organizations may also promote GPs from using guidelines in daily practice.

There was a widely shared belief in our study that many guidelines often answer general questions but are less likely to answer specific clinical questions in general practice, which is consistent with previous studies [29]. Constantly changing evidence of guidelines was recognized as another research barrier hindering the practice of EBM in our study. Therefore, developing more guidelines for GPs is urgently needed. Groups developing guidelines should ask relevant clinical questions and develop implementable and context-specific recommendations for GPs. Developers should be explicit and consistent in the development and presentation of recommendations.

### Strengths and limitations

To our knowledge, this is the first study assessing the behaviour of Chinese primary care providers who are incorporating EBM into their daily encounters with patients. Focusing on both the obstacles and needs for the application of EBM provides important clues and directions to integrate evidence-based information into primary care. However, some limitations need to be considered. The GPs in our study were selected from GPs who were master’s degree candidates in general practice, which calls into the question the generalizability of our results to other primary care providers. The majority of GPs in our study were female, whose views were not representative for all GPs in community. Accordingly, further qualitative studies are needed with larger sampling sizes and other primary care providers in China.

## Conclusions

The results of our study revealed GPs’ perceptions towards and behaviours of EBM and elicited the barriers and needs of GPs practising EBM. This qualitative study provided insight from GPs about the behaviours, barriers and needs of practising EBM. Using guidelines for chronic disease management is the most common EBM behaviour of GPs. GPs face many barriers in practising EBM, including patients’ poor compliance, lack of time, lack of resources, inadequate skills or knowledge, and guideline production problems. More clinical evidence-based guidelines, more sustainable training for improving EBM knowledge and skills, more resource support, and lesser workloads should be developed and implemented for GPs.

## Supplementary information

**Additional file 1.** Interview guides. A translated edition of the interview guides for four focus groups with the themes concerning GPs’ understanding of EBM, their clinical behaviour regarding EBM, existing barriers to implementing EBM, and needs of EBM.

## Data Availability

Transcripts will not be shared to protect the anonymity of the GPs. Readers who wish to gain access to the data can write to the corresponding author; data may be granted upon reasonable request.

## Abbreviations

CHSCs: Community health service centres
EBM: Evidence-based medicine
GP: General practitioner
