# Supplementary material for: Perceptions, behaviours, barriers and needs of evidence-based medicine in primary care in Beijing: a qualitative study
Source: BMC Fam Pract. 2019 Dec 6;20:171. doi: 10.1186/s12875-019-1062-0 (PMC6896763; doi:10.1186/s12875-019-1062-0)
Supplement: Supplementary file 1 — Additional file 1. Interview guides. A translated edition of the interview guides for four focus groups with the themes concerning GPs’ understanding of EBM, their clinical behaviour regarding EBM, existing barriers to implementing EBM, and needs of EBM. [file 12875_2019_1062_MOESM1_ESM.docx]

**Interview guides**

1. How do you think about evidence-based medicine?

2. What evidence-based medicine behaviours have you practiced?

3. What difficulties have you encountered when practicing EBM?

4. What further support do you need in order to practicing EBM better for patients?
